# Maintaining flippase activity in procoagulant platelets is a novel approach to reducing thrombin generation

**DOI:** 10.1111/jth.15641

**Published:** 2022-01-30

**Authors:** Sarah L. Millington‐Burgess, Matthew T. Harper

**Affiliations:** ^1^ 2152 Department of Pharmacology University of Cambridge Cambridge UK

**Keywords:** blood platelets, pharmacology, phosphatidylserine, phospholipid transfer proteins, thrombosis

## Abstract

**Background:**

During thrombosis, procoagulant platelets expose phosphatidylserine (PS), which enhances local thrombin generation. Reducing platelet PS exposure could be a novel anti‐thrombotic approach. PS is confined to the inner leaflet of the plasma membrane in unstimulated platelets by ATP‐dependent “flippase” activity. Ca^2+^ ionophores trigger all platelets to expose a high level of PS by activating a scramblase protein and inactivating the flippase. Although R5421 was previously shown to reduce Ca^2+^ ionophore‐induced PS exposure, its mechanism of action is unknown.

**Objectives:**

To determine the mechanism by which R5421 reduces platelet PS exposure.

**Methods:**

Washed human platelets were stimulated with the Ca^2+^ ionophore, A23187, to induce procoagulant platelet formation while bypassing proximal receptor signalling. Platelets PS exposure was detected using annexin V or lactadherin in flow cytometry. NBD (7‐nitro‐2‐1,3‐benzoxadiazol‐4‐yl)‐PS was used to assess scramblase and flippase activity. Thrombin generation was monitored using a fluorogenic substrate.

**Results and conclusions:**

R5421 reduced the extent of A23187‐stimulated platelet PS exposure, as demonstrated with annexin V or lactadherin binding. R5421 also maintained flippase activity in procoagulant platelets. Although R5421 appeared to inhibit scramblase activity in procoagulant platelets, it did not once the flippase had been inhibited, demonstrating that scramblase activity is not directly inhibited. Furthermore, R5421 inhibited the contribution of A23187‐stimulated platelets to thrombin generation. Together these data demonstrate that R5421 reduces the extent of PS exposure in procoagulant platelets by maintaining flippase activity. Maintaining flippase activity in procoagulant platelets is a novel and effective approach to reducing thrombin generation.

**Essentials**

- Platelet phosphatidylserine (PS) exposure promotes thrombosis by increasing thrombin generation.
- R5421 is a drug that inhibits platelet PS exposure but its mechanism was previously unclear.
- R5421 inhibits platelet PS exposure by maintaining the activity of a “flippase” protein.
- Regulating flippase activity may be a novel way to reduce thrombin generation and thrombosis.

## 1 INTRODUCTION

In a growing thrombus, a subset of activated platelets become procoagulant. These platelets expose phosphatidylserine (PS) on their surface and release PS‐positive extracellular vesicles (PS^+^‐EVs). PS binds circulating coagulation factors and enhances thrombin generation and occlusive thrombus formation.^1^ In unstimulated platelets, PS is confined to the inner leaflet of the plasma membrane (PM) because outward movement of PS is opposed by a “flippase.” The identity of this protein in platelets is unclear, but is likely to be a P4‐ATPase similar to ATP11C, which is the flippase in erythrocytes.^2^ Platelet flippase activity is inhibited by high cytosolic Ca^2+^ concentration ([Ca^2+^]cyt). In procoagulant platelets, a large rise in [Ca^2+^]cyt inactivates the flippase and activates a Ca^2+^‐dependent “scramblase,” TMEM16F, allowing PS to move to the outer leaflet unopposed.^3^, ^4^ PS exposure in activated platelets is normally considered to be an “all‐or‐nothing” event: activated platelets are either procoagulant with active scramblase, inactive flippase, and a high PS exposure; or non‐coagulant with inactive scramblase, active flippase, and no PS exposure.^5^, ^6^, ^7^

Inhibiting platelet PS exposure is a potential anti‐thrombotic strategy. We have previously shown that R5421 (ethaninidothioic acid), a putative scramblase inhibitor,^8^ inhibits platelet PS exposure in response to a Ca^2+^ ionophore, though R5421 also affects other platelet functions.^9^ However, here we demonstrate a novel anti‐thrombotic mechanism of action for R5421. Rather than inhibiting scramblase activity, R5421 reduces the extent of PS exposure in procoagulant platelets by maintaining flippase activity. Furthermore, we demonstrate that maintaining flippase activity in procoagulant platelets is a novel approach to reducing thrombin generation.

## 2 METHODS

### 2.1 Platelet preparation and monitoring of PS exposure by flow cytometry

Washed human platelets were isolated as previously described.^10^ Blood donors gave informed consent in accordance with the Declaration of Helsinki and with approval of the Human Biology Research Ethics Committee, University of Cambridge. Platelets were incubated with R5421 (Endotherm; 50 μM, 37°C, 60 min) or 0.1% DMSO (control), before stimulation with the Ca^2+^ ionophore, A23187 (10 μM, 10 min; 2 mM CaCl_2_ added).^8^ Platelets were stained with PE‐Cy7‐conjugated anti‐CD41 antibody (1:100; eBioscience 25‐0419‐42) to identify platelet events, and either FITC‐conjugated annexin V (AV; 1:100; eBioscience) or FITC‐conjugated lactadherin (50 μM; Haematologic Technologies) to measure PS exposure. Samples were fixed using 1% paraformaldehyde and diluted 1:1 in HEPES‐buffered saline (HBS) before analysis by flow cytometry. PE‐Cy7 fluorescence was used to trigger event acquisition. Platelets and PS^+^‐EVs were gated on their forward scatter (FSC) profiles. High AV or lactadherin binding was gated such that >98% of DMSO‐treated A23187‐stimulated platelets were included. Most unstimulated platelets show low binding (AV or lactadherin negative), with the gate set such that 1% of DMSO‐treated unstimulated platelets bound AV or lactadherin.

Phosphatidylcholine (PC):PS liposomes (6% PS) were generated by sonication. Lipids were hydrated in distilled water and vortexed to achieve a cloudy suspension, then sonicated until a clear liposome solution formed. Liposomes were stained with FITC‐conjugated AV (1:100) in the presence of 2.5 mM Ca^2+^ then analyzed by flow cytometry.

Where required, platelets were loaded with Fluo‐5N as previously described.^6^

### 2.2 Flippase activity

Platelets were incubated with 5 mM N‐ethylmaleimide (NEM) or HBS (control) for 10 min before incubation with R5421 or DMSO. Platelets were then stimulated with 10 μM A23187 (5 min) before incubation with 0.5 µM NBD (7‐nitro‐2‐1,3‐benzoxadiazol‐4‐yl)‐PS (10 min). Platelets were then transferred to an equal volume of HBS ± BSA (bovine serum albumin; 2% w/v; 5 min), stained with PE‐Cy7‐CD41a (2 min) and fixed with 1% paraformaldehyde. Samples were analyzed by flow cytometry and the proportion of non‐BSA extractable NBD fluorescence calculated.

### 2.3 Scramblase activity

Platelets were loaded with 0.5 µM NBD‐PS (45 min; 30°C), then incubated with 5 mM NEM or HBS (control) for 10 min. Platelets were then incubated with R5421 or DMSO (control) and then stimulated with 10 μM A23187 (10 min). Platelets were transferred to an equal volume of HBS ± BSA for 5 min, stained with PE Cy7‐CD41a for 2 min, and fixed with 1% paraformaldehyde. Samples were analyzed by flow cytometry and non‐BSA extractable NBD‐PS fluorescence calculated.

### 2.4 Thrombin generation

Washed platelets were treated with R5421 or DMSO (control). HBS was then added 1:1 (v/v) and supplemented with 100 nM prostaglandin E_1_ (PGE_1_) and apyrase (grade VII; 0.02 U/ml). Platelets were pelleted by centrifugation (600 g, 10 min, ambient temperature) and then resuspended in autologous platelet‐poor plasma (filtered to remove extracellular vesicles >0.22 μm), at 1.5 × 10^8^ platelets/ml. Platelet‐rich plasma was stimulated with 10 μM A23187 (10 min). Thrombin generation was triggered with 16.6 CaCl_2_ ± 5 pM tissue factor. Cleavage of the substrate Z‐Gly‐Gly‐Arg‐AMC HCl was measured by the calibrated automated thrombogram method in a FLUOStar Omega Microplate Reader at 37°C. Thrombin concentrations were determined using a Thrombin Calibrator (Stago) following correction for the inner filter effect and substrate consumption.^11^

## 3 RESULTS AND DISCUSSION

**FIGURE 1:**
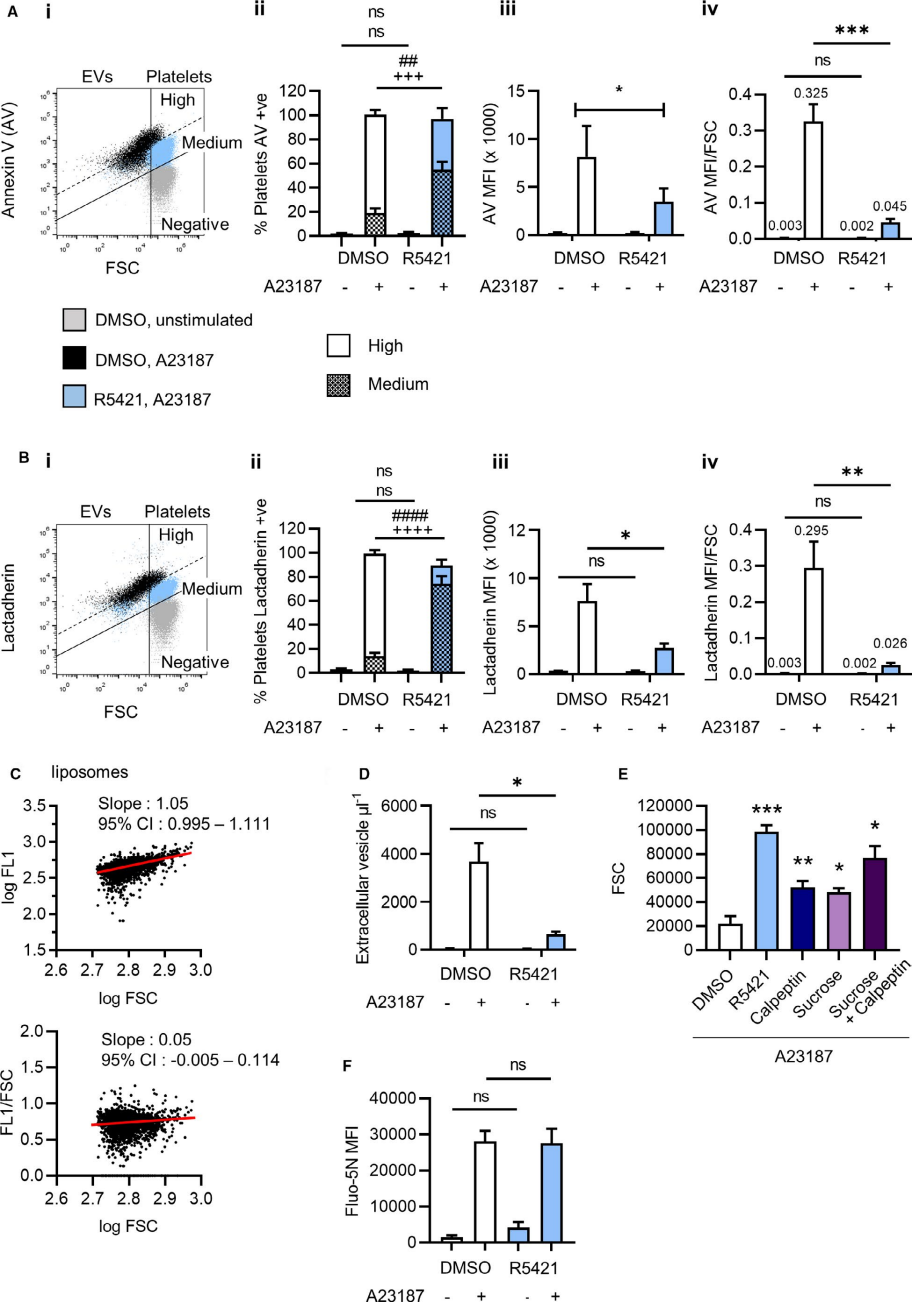
R5421 reduces the extent of platelet phosphatidylserine (PS) exposure. Washed human platelets were incubated with 50 μM R5421 for 60 min before stimulation with 10 μM A23187 in the presence of 2 mM Ca^2+^ for 10 min, all at 37°C. Platelets were labelled with (A) FITC‐conjugated annexin V (AV) or (B) FITC‐conjugated lactadherin, fixed in 1% paraformaldehyde and analyzed by flow cytometry. i, Representative flow cytometry dot plots showing DMSO‐treated unstimulated platelets (gray), DMSO‐treated A23187‐stimulated platelets and extracellular vesicles (EVs; black), and R5421‐treated A23187‐stimulated platelets and extracellular vesicles (blue). Platelets were gated from extracellular vesicles based upon their forward scatter (FSC) profile. “Medium” and “high” levels of AV binding to stimulated platelets are indicated. ii, Data analyzed as % platelets (i.e., excluding EVs) with high or medium levels of AV binding (Aii) or lactadherin binding (Bii). iii, Data analyzed as median fluorescent intensity (MFI) of platelets (i.e., excluding EVs). iv, Data analyzed as MFI of platelets divided by median FSC. Data show mean ± standard error of the mean, *n* = 6, DMSO‐treated platelets—white bars, R5421‐treated platelets—blue bars. All data analyzed using 2‐way analysis of variance (ANOVA), both factors matching and Sidak’s multiple comparisons test. ns, not significant; *P < .05, **P < .01, ***P < .001. ##*P* < .01, ####*P* < .0001 comparing high AV or lactadherin; +++*P* < .001; ++++*P* < .0001 for medium AV or lactadherin binding. C, Phosphatidylcholine (PC):PS liposomes were stained with AV‐FITC in the presence of 2.5 mM CaCl_2_. In the upper panel, log AV‐FITC fluorescence is plotted against log FSC. Logarithmic scales were used as these are also normally used for platelet AV binding. Linear regression showed increasing log AV‐FITC fluorescence with increasing log FSC, despite the liposomes having the same % PS. In the lower panel, the AV‐FITC fluorescence (FL1) of each liposome was divided by its FSC. Following normalization there was no linear relationship between FL1/FSC and log FSC, as shown by a linear regression slope not significantly different to zero. D, 60‐min incubation with R5421 inhibits release of PS^+^ extracellular vesicles. E, A 60‐min incubation with R5421 prevents the FSC changes in procoagulant platelets. Calpeptin (140 μM, 30 min) also partially prevents FSC changes, as does a 200 mM hypertonic sucrose solution added prior to A23187 stimulation. *N* = 3–5. Data analyzed using repeated measures 2‐way ANOVA, and Sidak’s multiple comparisons test. ns, not significant; *P < .05, **P < .01, ***P < .001, ****P < .0001 compared to DMSO‐treated platelets. F, Platelets were loaded with low‐affinity fluorescent Ca^2+^ dye, Fluo‐5N, during treatment with R5421 (or DMSO) then stimulated with A23187. Fluo‐5N MFI was monitored by flow cytometry. *N* = 3; ns, not significant

Procoagulant platelet formation was triggered in platelets by stimulation with the Ca^2+^ ionophore, A23187. A Ca^2+^ ionophore was used to bypass signalling immediately downstream of cell surface receptors, as we have previously shown that these signals are disrupted by R5421.^9^ As expected, A23187 triggered high AV binding to almost all platelets, indicating a high level of PS exposure. R5421 reduced the extent of AV binding in response to A23187, such that the percentage of platelets with “high” AV binding was significantly inhibited, as previously reported.^9^ In contrast, the percentage with “medium” AV binding was significantly increased (Figure 1Ai–ii), and the total percentage of platelets with AV binding higher than unstimulated platelets was not significantly affected, indicating that all platelets had become procoagulant but with a lower extent of PS exposure. These data demonstrate that platelet PS exposure is not an “all‐or‐nothing” event. This was also indicated by reduced median fluorescence intensity (MFI) of bound AV‐FITC (Figure 1Aiii). We confirmed this with lactadherin‐FITC, which is more sensitive to lower levels of membrane PS.^12^ R5421 pre‐treatment substantially inhibited lactadherin‐FITC MFI and almost all platelets showed medium lactadherin‐FITC binding (Figure 1Bi–iii). The reduction in MFI of AV‐FITC and lactadherin‐FITC was not due to fluorescence quenching by R5421 (data not shown).

R5421 also prevented the characteristic reduction in FSC seen in procoagulant platelets compared to unstimulated platelets (Figure 1Ai,Bi).^13^ It is possible that the change in FSC may complicate the interpretation of AV or lactadherin binding. FSC is often interpreted as cell size. Larger platelets might be expected to bind more AV or lactadherin than smaller platelets despite the same percentage of PS in their outer membrane. Similarly, in PC:PS liposomes (6% PS), higher AV binding was seen to liposomes with greater FSC (Figure 1C). This effect can be corrected by dividing AV‐FITC MFI by liposome FSC. Applying this to platelets, AV‐FITC MFI/FSC and lactadherin‐FITC MFI/FSC ratios were significantly inhibited by R5421 (Figure 1Aiv,Biv). The decrease in FSC in procoagulant platelets may be due to release of PS^+^‐EVs. Notably, R5421 inhibited PS^+^‐EV release from A23187‐stimulated platelets (Figure 1D). Moreover, calpeptin, a calpain inhibitor, also inhibited PS^+^‐EV release and partially inhibited the reduction in FSC (Figure 1E). The decrease in FSC may also result from changes in light refraction due to “ballooning” in procoagulant platelets. Platelet ballooning requires extensive PS exposure and does not occur in the absence of TMEM16F.^14^ Hypertonic sucrose (200 mM), which inhibits ballooning,^15^ also partially prevented the reduction in FSC (Figure 1E). Calpeptin and hypertonic sucrose together inhibited the FSC reduction to a greater extent than either alone. Together, these data indicate that R5421 does inhibit PS exposure in procoagulant platelets, despite no difference in total percentage AV positive. Rather than inhibiting procoagulant platelet formation, R5421 reduces the extent of PS exposure, such that R5421‐treated procoagulant platelets show medium PS exposure compared to high PS exposure in DMSO‐treated procoagulant platelets. Moreover, although the extent of PS exposure, measured by flow cytometry, may be obscured by other PS‐dependent events in procoagulant platelets, such as ballooning and release of PS^+^‐EVs, R5421 substantially reduced AV or lactadherin binding even once the fluorescence had been normalized to FSC. Together, these data show that R5421 treatment reduces the extent of PS exposure in procoagulant platelets.

We previously reported that R5421 does not affect A23187‐induced [Ca^2+^]cyt signalling.^9^ However, this was with a high‐affinity Ca^2+^ indicator that may be saturated by the high [Ca^2+^]cyt in procoagulant platelets.^6^ We therefore monitored Ca^2+^ signalling in platelets loaded with the low affinity Ca^2+^ indicator, Fluo‐5N. A23187‐induced Fluo‐5N fluorescence was not affected by R5421 (Figure 1F).

**FIGURE 2:**
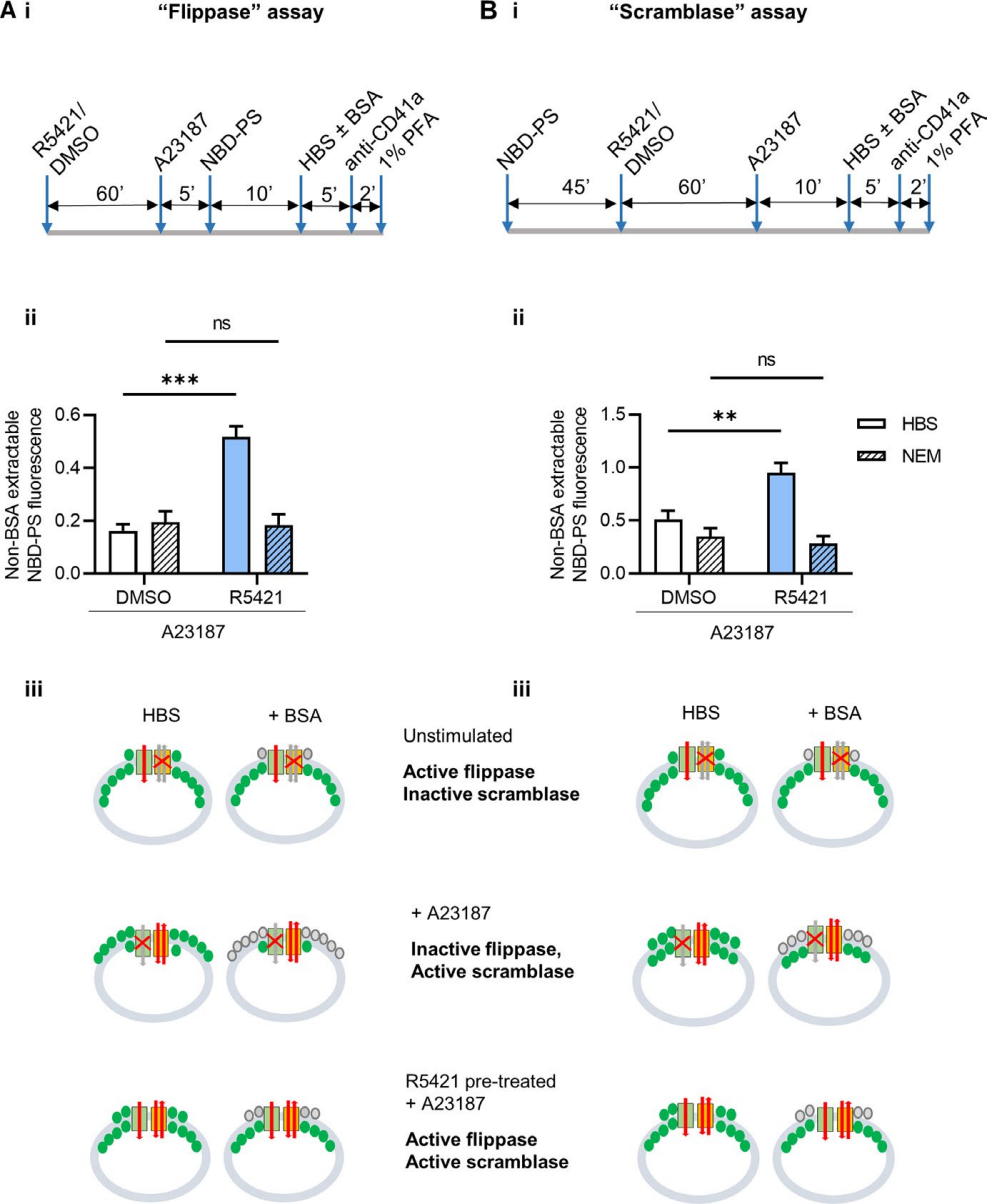
R5421 maintains flippase activity in procoagulant platelets. Washed human platelets were tested in a “flippase activity” assay (A) or a “scramblase activity” assay (B). The experimental protocols are shown in (i). ii, Non‐bovine serum albumin extractable NBD(7‐nitro‐2‐1,3‐benzoxadiazol‐4‐yl)‐PS (phosphatidylserine) fluorescence following a “flippase assay” or “scramblase assay” in A23187‐stimulated platelets, with a 60‐min incubation with DMSO or R5421 and pre‐treated with 5 mM NEM (N‐ethylmaleimide; hatched bars). Data show mean ± standard error of the mean, *n* = 4, DMSO‐treated platelets—white bars, R5421‐treated platelets—blue bars. Data analyzed using 2‐way analysis of variance, both factors matching and Sidak’s multiple comparisons test. ns, not significant, **P < .01, ***P < .001. iii, Schematic diagram showing flippase activity, scramblase activity, and PS levels in unstimulated platelets, procoagulant platelets (A23187‐stimulated) and R5421 pre‐treated activated procoagulant platelets. PS is shown in green. Flippase and scramblase activity are shown using red arrows. Inactive pathways shown using gray arrows and red cross

Having established that R5421 inhibits the extent of PS exposure, we next investigated its mechanism of action using fluorescent NBD‐PS. NBD‐PS can be extracted from the PM outer leaflet by BSA. The proportion of NBD‐PS fluorescence that cannot be extracted gives a measure of PS in the inner leaflet. Assays using NBS‐PS have been previously used to monitor flippase and scramblase activity.^3^ The assay protocols are shown in Figure 2. In some experiments, platelets were pre‐treated with the flippase inhibitor, NEM. In a “flippase assay,” platelets were treated with R5421 or DMSO, stimulated with A23187, then NBD‐PS was added. Upon stimulation with A23187, the flippase would be expected to be inactivated in procoagulant platelets and little NBD‐PS subsequently taken into the PM inner leaflet resulting in low non‐BSA extractable fluorescence. However, in R5421‐treated platelets, non‐BSA extractable fluorescence remained high after A23187 stimulation. In contrast, when flippase activity was inhibited by NEM, flippase activity was no longer maintained in R5421‐treated platelets (Figure 2A). Together, these data suggest that the flippase remains active in R5421‐treated procoagulant platelets.

To complete a “scramblase assay” (Figure 2B), platelets were pre‐loaded with NBD‐PS (which moves to the inner leaflet by flippase activity). Platelets were then stimulated with A23187, activating the scramblase. Unopposed by flippase activity (normally inactive in procoagulant platelets), scramblase activity results in NBD‐PS being exposed to the outer leaflet, where it is extracted by BSA. Non‐BSA‐extractable fluorescence is therefore low. This “scramblase activity” appeared to be inhibited by R5421. However, this “scramblase assay” measures the net movement of PS to the PM outer leaflet and the above interpretation assumes that the flippase is inactive in procoagulant platelets. If, however, the flippase remains active then net outward PS movement would reduce. In this assay, it would *appear* as if scramblase activity were reduced. Importantly, when flippase activity was inhibited by NEM before incubation with R5421 and stimulation with A23187, “scramblase activity” was no longer inhibited by R5421. This indicates that the perceived inhibition of scramblase activity is actually due to the flippase remaining active, reducing the net NBD‐PS in the outer leaflet that could be extracted by BSA. Taken together, these results demonstrate that R5421 is not a scramblase inhibitor. Instead, to the best of our knowledge, it is the first identified compound that maintains flippase activity in procoagulant platelets.

**FIGURE 3:**
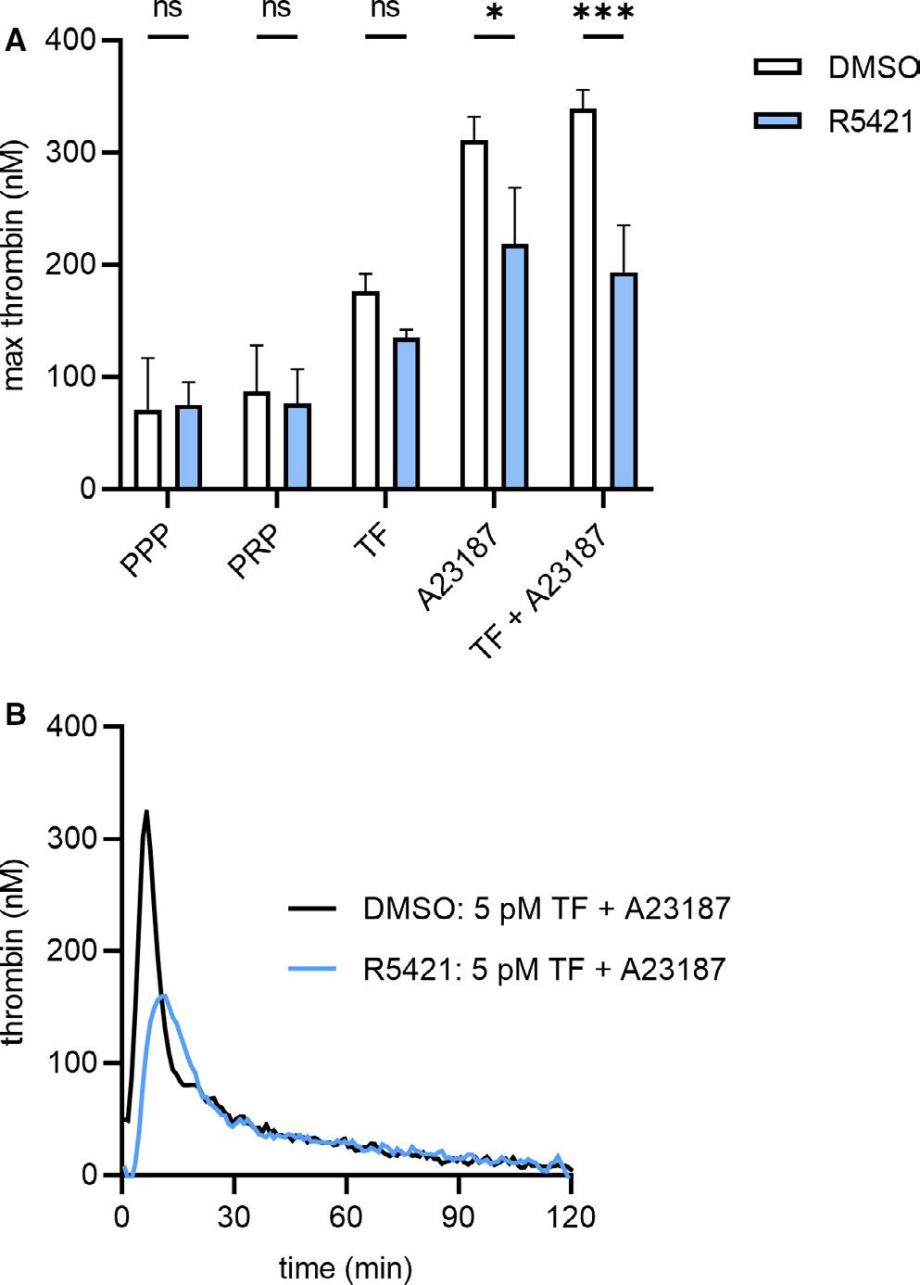
R5421 inhibits thrombin generation. Washed human platelets were incubated with R5421 for 60 min at 37°C and then washed and re‐suspended in filtered autologous platelet‐poor plasma (PPP) to 1.5 × 10^8^ platelets/ml. A, maximum thrombin generation in PPP, platelet‐rich plasma (PRP), or PRP in the presence of 5 pM tissue factor (TF), 10 μM A23187, or both TF and A23187. B, Representative thrombin generation traces of DMSO or R5421 pre‐treated platelets and stimulation with 10 μM A23187 in the presence of 5 pM TF. Data show mean ± standard error of the mean, *n* = 4, DMSO‐treated platelets—white bars, R5421‐treated platelets—blue bars. Data analyzed using repeated measures 2‐way analysis of variance, and Sidak’s multiple comparisons test. ns, not significant, *P < .05, ***P < .001

Finally, to assess the functional relevance of flippase modulation and the reduced extent of PS exposure on platelets, we tested the effect of R5421 on thrombin generation. R5421 appears ineffective in plasma (data not shown), but because the effect of R5421 is irreversible (Dekkers et al.^8^ and confirmed by us; data not shown), platelets were incubated with R5421, washed, and re‐suspended in autologous platelet‐poor plasma. Strongly activated procoagulant platelets enhanced thrombin generation. R5421 inhibited the contribution of A23187‐stimulated platelets to thrombin generation, even in the presence of a high concentration of tissue factor (5 pM; Figure 3). R5421 therefore inhibits the contribution of procoagulant platelets to thrombin generation.

**FIGURE 4:**
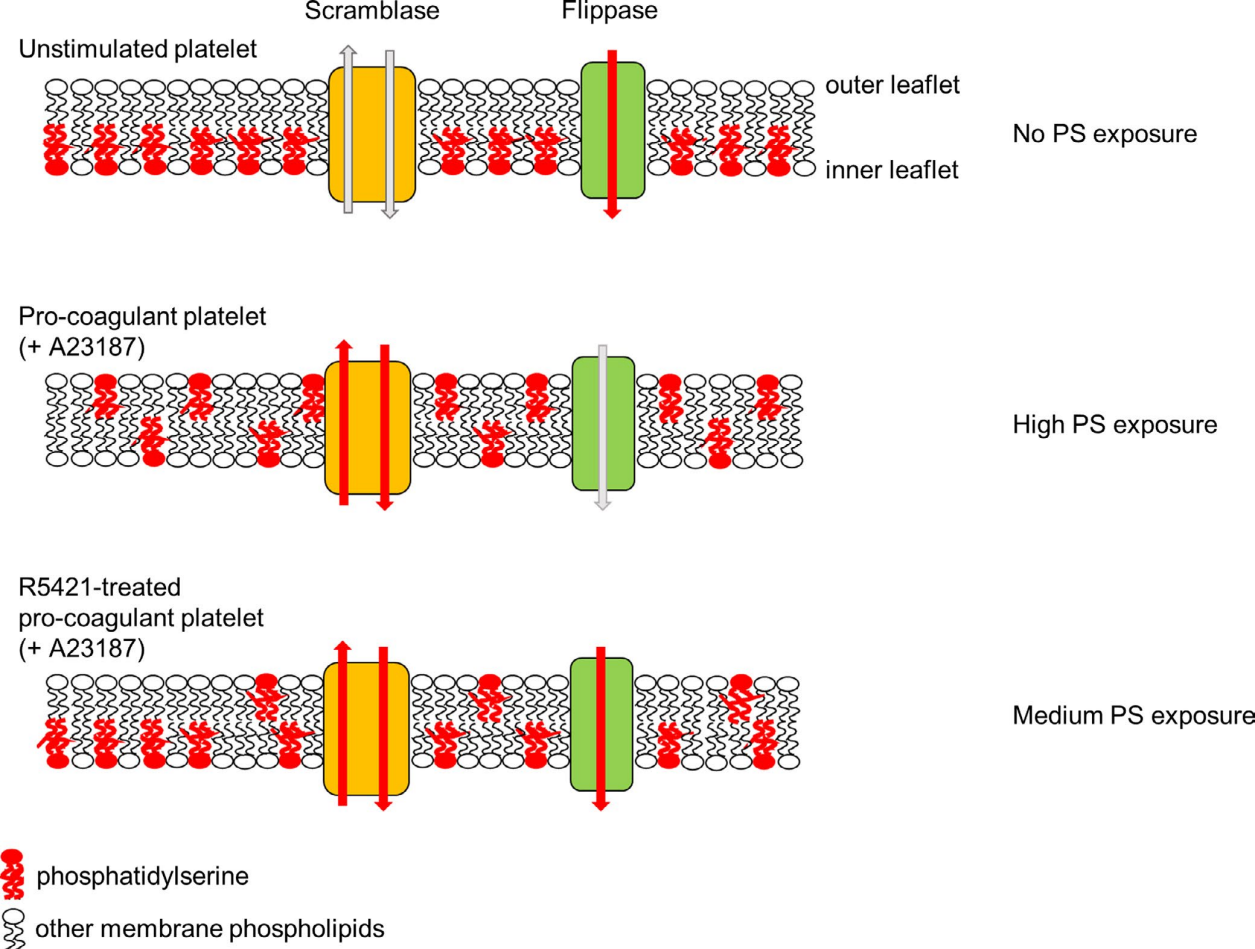
Proposed model of differing levels of phosphatidylserine (PS) exposure. In unstimulated platelets, active flippase restricts PS to the inner leaflet. In procoagulant platelets (e.g., following A23187 stimulation), active scramblase and inactive flippase results in loss of membrane asymmetry and high PS exposure. However, in R5421‐treated platelets, flippase is still active, reducing the net movement of PS to the outer leaflet. The result is a lower level of PS exposure and reduced thrombin generation

This study identifies R5421 as the first compound known to maintain flippase activity in procoagulant platelets (Figure 4), which reduces the extent of PS exposure and inhibits thrombin generation. Although the flippase is normally inactivated by high [Ca^2+^]cyt in procoagulant platelets, R5421 prevents flippase inactivation in response to this Ca^2+^ signal. Whether R5421 maintains flippase activity in PS‐exposing apoptotic platelets is not yet clear, though we have previously shown that R5421 inhibits AV binding in platelets induced to undergo apoptosis by the BH3 mimetic, ABT‐737.^16^ Although further work is required to identify the flippase protein in platelets, understand its regulation, and develop novel compounds that modulate its activity in a more selective manner than R5421, this study provides proof of principle for the utility of such an approach.

## CONFLICTS OF INTEREST

The authors declare that there are no scientific or financial conflicts of interest relating to this work.

## AUTHOR CONTRIBUTIONS

SLB designed research, performed experiments, analyzed data, and co‐wrote the manuscript. MTH designed research and co‐wrote the manuscript.
